# Laser-induced etching of few-layer graphene synthesized by Rapid-Chemical Vapour Deposition on Cu thin films

**DOI:** 10.1186/2193-1801-1-52

**Published:** 2012-11-27

**Authors:** Marco Piazzi, Luca Croin, Ettore Vittone, Giampiero Amato

**Affiliations:** 1 Quantum Research Laboratory, Istituto Nazionale di Ricerca Metrologica, Strada delle Cacce 91, 10135 Turin, Italy; 2 Department of Physics, NIS Centre of Excellence and CNISM, University of Turin, Via Pietro Giuria 1, 10125 Turin, Italy; 3 Department of Applied Science and Technology, Politecnico of Turin, Corso Duca deli Abruzzi 24, 10129 Turin, Italy

**Keywords:** CVD graphene, Copper, Laser induced etching, Heating ablation effects, 61.80.Ba, 81.15.Gh, 61.48.Gh, 81.05.ue

## Abstract

The outstanding electrical and mechanical properties of graphene make it very attractive for several applications, Nanoelectronics above all. However a reproducible and non destructive way to produce high quality, large-scale area, single layer graphene sheets is still lacking. Chemical Vapour Deposition of graphene on Cu catalytic thin films represents a promising method to reach this goal, because of the low temperatures (*T* < 950°C−1000°C) involved during the process and of the theoretically expected monolayer self-limiting growth. On the contrary such self-limiting growth is not commonly observed in experiments, thus making the development of techniques allowing for a better control of graphene growth highly desirable. Here we report about the local ablation effect, arising in Raman analysis, due to the heat transfer induced by the laser incident beam onto the graphene sample.

## Background

Graphene (a single bidimensional layer of carbon atoms arranged in an hexagonal lattice) has attracted a major interest in the last few years because of its astonishing electrical (Castro Neto et al. 2009; Peres 2010; Peres et al. 2006), mechanical (Lee at al. 2008) and chemical properties (Elias et al. 2009; Wang et al. 2009a), that make it a good candidate for the future development of nanoelectronics devices. Although the main properties of this material are nowadays well known from a theoretical point of view, an efficient and highly reproducible method to grow high quality, large-scale area, single layer graphene films, suitable for practical applications, is still lacking. For this reason, several techniques have been developed in the last years in order to achieve this goal: the most important are the epitaxial growth of graphene by thermal sublimation of SiC (de Heer et al. 2007; Emtsev et al. 2009; Hass et al. 2008; Sprinkle et al. 2009; Varchon et al. 2007), the Chemical Vapour Deposition (CVD) synthesis of graphene on various metal catalysts (Reina et al. 2009; Lee et al. 2010; Liu et al. 2010; Kim et al. 2012; Nandamuri et al. 2010; Somani et al. 2006; Li et al. 2009a; 2009b; Tao et al. 2012) and the chemical reduction of graphene oxide (Gilje et al. 2007; Lee at al. 2009; Paredes et al. 2008; Schniepp et al. 2006). Among these, CVD technique seems to be one of the most promising methods because of the reported possibility (Liu et al. 2010) of obtaining highly uniform, defect-free graphene flakes as large as ∼ 100*μ*m^2^ in a reproducible, highly accessible and inexpensive way.

Since CVD synthesis needs a catalyst to activate the chemical decomposition of the carbon precursor (usually methane or ethylene) used for graphene growth at low temperatures (*T* < 950°C – 1000°C), the use of many metals (Ir (Coraux et al. 2008), Ru (Martoccia et al. 2008), Pt (Sasaki et al. 2000; Starr et al. 2006), Fe (Kondo et al. 2010), Ag (Di et al. 2008), Ni (Liu et al. 2010; Kim et al. 2009; Obraztsov et al. 2007), Cu (Bae et al. 2010; Li et al. 2009a; Tao et al. 2012) as catalysts during the process has been reported in literature. Cu is one of the most promising catalyst (Mattevi et al. 2011) because of the low C solid solubility in it (0.001−0.008 weight % at ∼ 1084°C): this property brings to the formation of only soft (not covalent) bonds between the *Π* electrons of 2p_*z*_orbitals of sp^2^ hybridized C atoms and the 4s electrons of Cu, without formation of any carbide phase during the growth process. As a consequence, formation of graphene should stop after one single layer has been formed: this makes CVD growth of graphene on Cu very attractive. Nonetheless, many experiments show that actually such a self-limiting behaviour is hardly observed, since few-layered graphitic structures are usually grown on Cu substrates.

For this reason, to obtain monolayer graphene, several post processing techniques have been proposed to selectively etch atomic graphene layers. Among the various approaches (e.g. heat-induced etching by oxygen (Liu et al. 2008), e-beam lithography assisted technique (Novoselov et al. 2004; Zhang et al. 2005), graphene cutting by carbon-soluble metals (Campos et al. 2009; Datta et al. 2008) the thinning of atomic carbon multilayers by laser irradiation (Han et al. 2011) can be a promising method to obtain monolayer graphene. In this last work, authors show how the central rôle in graphene etching is held both by the laser irradiation used for the Confocal Raman Spectroscopy performed on the samples, and by the SiO_2_/Si substrate on top of which few-layered graphene has been transferred: the heat produced by the irradiation “burns” (in presence of oxygen) locally the outermost C layers, while the innermost layer (the one bound to the substrate) is left unetched because of the presence of the SiO_2_ layer acting as heat sink.

Cu, being a metal, has thermal conductivity higher than SiO_2_ and can represent therefore an enhanced heat sink, so it can be expected to observe a similar behaviour also for graphene grown by CVD on it.

Here we report the change in shape and position of the G and 2D peaks observed in Raman spectra acquired at different time intervals on the same spot of a graphene sample synthesized by CVD on Cu thin films: the evolution of the spectra, indicating that the structure of graphene is changing during the exposure to the laser used during Raman analysis, is compatible with a decrease in the number of graphene layers present on the substrate. This decrease may be attributed to the same laser-induced etching effect observed on graphene deposited onto SiO_2_/Si substrates. If this is the case, an efficient and easy graphene etching technique can be developed and a promising way to obtain high uniform, large-scale area, monolayer graphene can be envisaged.

## Methods

### Cu deposition

The samples subjected to CVD process have been prepared by e-beam evaporation of 500 nm Cu thin film on top of a p-type (100) oriented Si wafer (∼ 1 cm^2^) with ∼ 300 nm thermal SiO_2_. The deposition has been carried out in a load-lock chamber at a base pressure of ∼ 10^−8^ mbar and deposition pressure of ∼ 10^−6^ mbar, with an average growth rate of 3−5 Å/s.

The thickness *d* of deposited Cu film has been chosen in order to limit the known problem (Mattevi et al. 2011) of dewetting occurring on very thin films (*d* < 500 nm) at temperatures ≳ 800°C.

**Figure 1:**
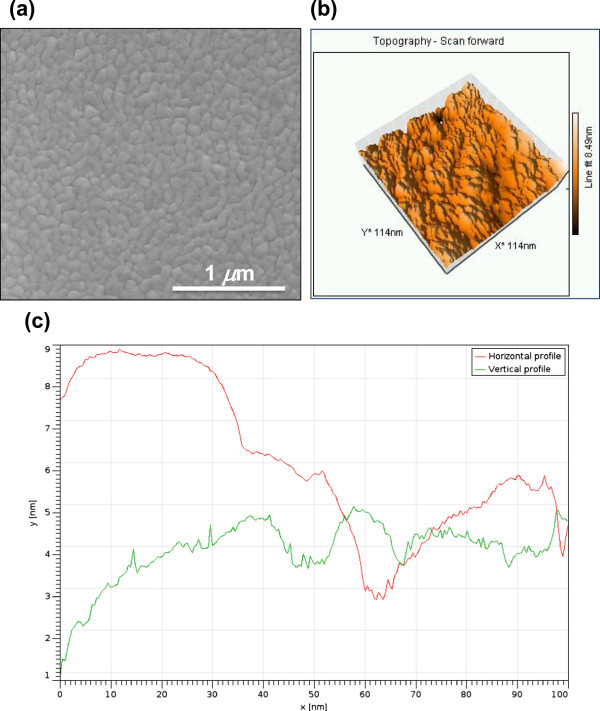
**SEM and STM Analyses of e-beam evaporated Cu thin films as deposited.** (**a**) SEM image of the polycrystalline structure of the Cu surface after e-beam evaporation; (**b**) STM topographic image of a small portion -∼ 10^−2^*μ*m^2^- of the Cu surface; (**c**) topographic profiles along two directions (red: horizontal, green: vertical) obtained with STM analysis.

Scanning Electron Microscopy (SEM) images of the samples (taken with a FEI *InspectF* Scanning Electron Microprobe) after Cu deposition show a uniform coverage of the SiO_2_ surface characterized by a Cu polycrystalline structure with grains of ∼ 90 nm as typical size (Figure 1(a)). Moreover, Scanning Tunneling Microscopy (STM) scanning an area of ∼ 10^−2^*μ*m^2^ allowed us to evaluate the roughness of the Cu surface (Figure 1(b)). The resulting root mean square (RMS) of ∼ 2 nm (an order of magnitude higher than single layer graphene thickness, ∼ 3.3 Å), together with the topographic behaviours obtained for certain scanning directions (Figure 1(c)), showing among others height variations as small as few angstroms, makes ineffective the use of Atomic Force Microscopy (AFM) to detect any change in the number of graphene layers eventually present on Cu: the changes in height produced by the latter effect would be hardly distinguishable from topographic changes due to the roughness of the substrate’s surface.

### CVD growth process

**Figure 2:**
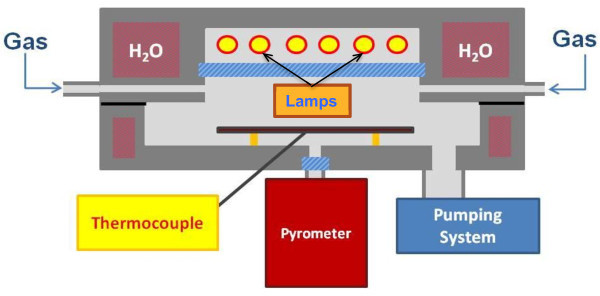
**RTA system for graphene deposition.** Schematic view of RTA system working principles: heating lamps provide heat only to the sample.

Before undergoing CVD process, samples have been carefully cleaned in acetone and isopropanol. CVD has been then performed in a Rapid Thermal Annealing (RTA) system (Jipelec *JetFIRST 100*) suitable for depositions in Low Vacuum conditions (*p*_min_ ∼ 10^−2^mbar) up to *T*_max_ ∼ 1300°C. The system is equipped with four gas lines controlled through mass flow meters and it is characterized by a small heat capacity allowing for fast cooling-down processes, up to ∼ 300°C/min (see Figure 2).

The temperature of the system is controlled by a pyrometer, exposed to the back of the sample holder (a 4” Si wafer) and calibrated by means of a thermocouple in contact with it (Figure 2). The pyrometer sets the power of the lamps. Since the Cu sample undergoing the CVD process is directly exposed to the lamps, the temperature reading by the pyrometer (correct in absence of Cu) is probably lower than the temperature reached by the surface during the process. Therefore, the real deposition temperature may be underestimated. We are performing some studies on this topic in order to solve this ambiguity in the future. However, at the temperature reached during the process as read by the pyrometer dissociation of CH_4_ and subsequent graphene formation take place, while the undesired Cu dewetting effect is prevented.

**Figure 3:**
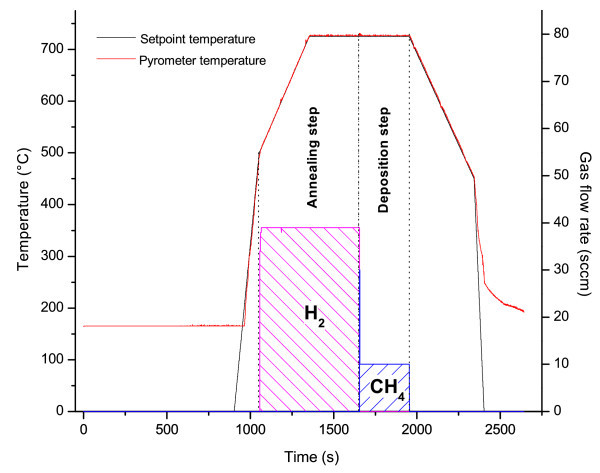
**Expected thermal cycle for graphene deposition.** Illustration of the thermal process performed for CVD growth of graphene on top of Cu: see text for details.

The deposition process started with a fast heating step, increasing the temperature up to 500°C, carried out in vacuum (*p*_chamber_ = 1.2·10^−2^ mbar). A subsequent annealing step (10 minutes long), bringing the system to 725°C, has been then performed under 40 sccm of H_2_ (*p*_chamber_ = 1.65 mbar). Purpose of this step is to improve the quality of the Cu film, by increasing the size of the grains present on its surface and, consequently, by inducing a low defective growth of graphene due to the reduced number of grain boundaries. The synthesis of graphene has been performed by flowing ultrahigh purity CH_4_, with a flow rate of 10 sccm, for 5 minutes (*p*_chamber_ = 3.3·10^−1^mbar), without any H_2_ flow, following (Tao et al. 2012) in which H_2_ has been reported to be detrimental for the final quality of graphene sheets when using Cu thin films. After deposition the system has been cooled-down to room temperature in three steps: a first one (at a rate of ∼ 42°C/min), down to 450°C in gas-free conditions, a faster one by switching the heaters off (at a rate of ∼ 180°C/min), till to ∼ 270°C, again in vacuum conditions and a final one carried out in nitrogen atmosphere, to purge the system, down to room temperature. The thermal cycle has been reported in Figure 3 for clarity.

### Graphene transfer

Graphene has been transferred from Cu to new insulating (285 nm-SiO_2_/Si) substrates by means of a standard chemical etching technique. Approximately 1.5 *μ*m of Poly(methyl-methacrylate) (PMMA) have been firstly deposited on graphene by spinning (at 5000 rpm for 1 minute) and heating (at 165°C for 5 minutes) the samples several times. After PMMA deposition, samples have been dipped in a 0.5 M diluted FeCl_3_ etching solution for ∼ 4 hours (keeping them at 45°C to enhance etching kinetics). Once Cu has been completely etched away from the samples, PMMA/graphene membranes have been picked up onto 285 nm-SiO_2_/Si substrates and PMMA has been finally removed with acetone.

## Results and discussion

Graphene samples have been first characterized by SEM analysis and X-Ray Diffraction (XRD, performed with a Philips *X’Pert Pro* X-ray system using Co radiation beam at 0.8° incident angle) in order to investigate a change in granulometry and crystallographic orientation of the Cu surface, due to high temperature annealing under hydrogen flow.

**Figure 4:**
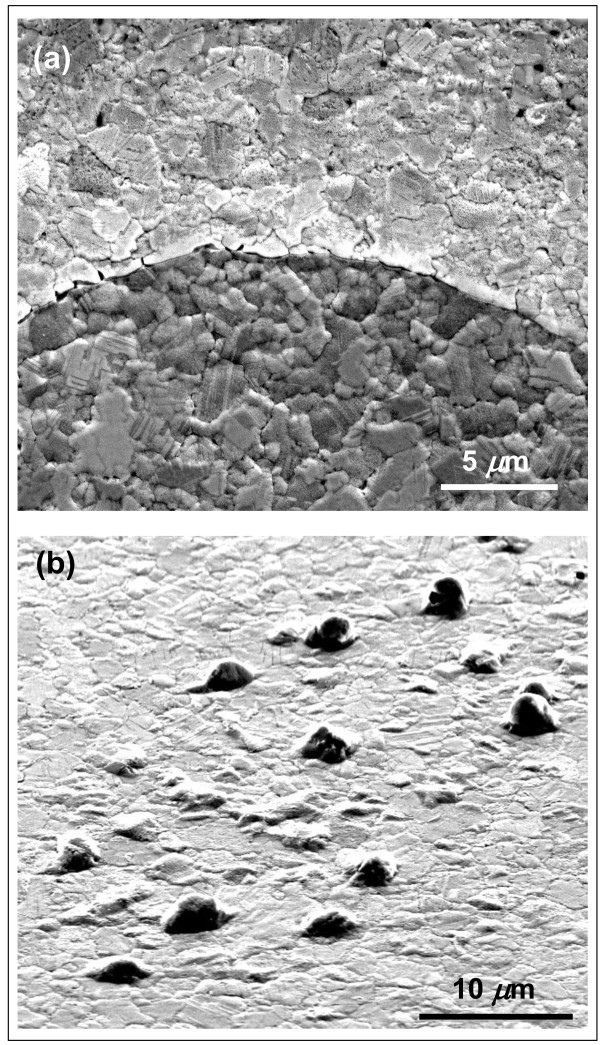
**SEM Analysis of Cu samples after graphene deposition.** SEM images of a Cu sample after CVD process: (**a**) Cu surface reorganizes itself by increasing the size of the grains; (**b**) Cu droplets start to appear on top of the surface.

SEM image in Figure 4(a) shows that Cu surface reorganized itself at the temperature reached during graphene deposition: an increase of grains average size (from ∼ 90 nm to ∼ 3*μ*m) is observed, as expected. Cu still covers uniformly the sample surface, meaning that the temperature during the annealing and deposition processes was low enough and the growth time short enough to prevent dewetting effects on the catalytic film. Nevertheless, early stage formation of Cu droplets is observed (Figure 4(b)).

**Figure 5:**
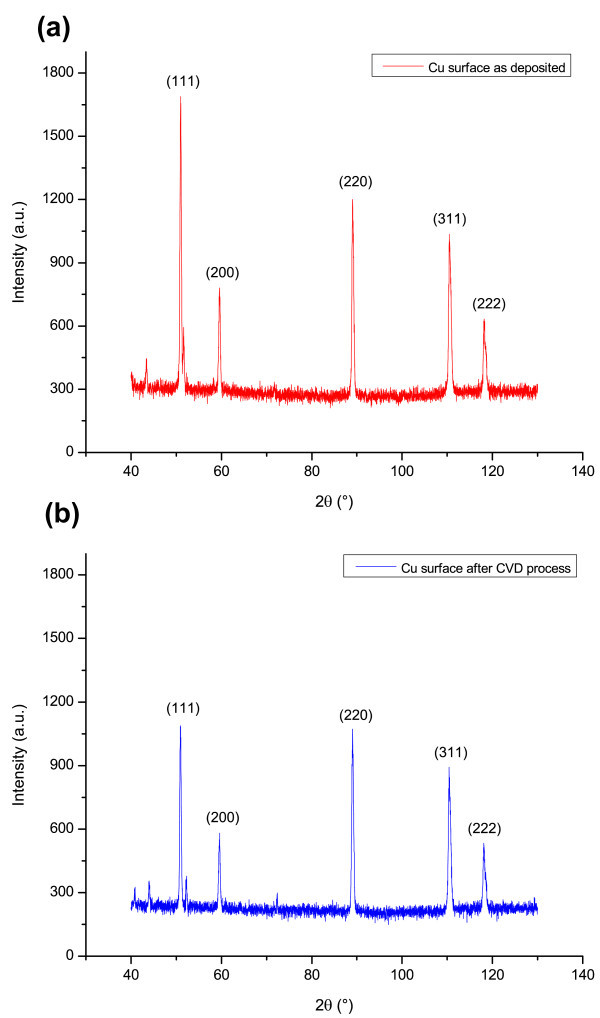
**XRD Analysis of Cu surface.** XRD analysis of the Cu surface: (**a**) as deposited after e-beam evaporation; (**b**) after high temperature treatment at 725°C.

XRD analysis performed on our samples, in contrast to what observed in other works (Tao et al. 2012), shows (Figure 5) changes in the preferential crystallographic orientation of the grains present on the sample surface after CVD process at the nominal temperature of 725°C, involving both (111) and (220) directions, with a decrease in the I_(111)_/I_(220)_ intensity ratio after the thermal treatment. This fact can probably be ascribed to the fast cooling-down rate chosen for the experiment.

Samples have been finally characterized with Raman spectroscopy by means of a Renishaw *In-Via Raman Microscope*, equipped with He-Cd blue laser at 442 nm, avoiding this way background plasmon emission of Cu when excited e.g. by green light (Tao et al. 2012). For the acquisition we have used laser power both at 1 mW and 0.1 mW, by varying consequently the exposure time of the samples to laser irradiation (in order to keep the amount of energy *E* = *Pt* transferred from laser light to samples fixed).

**Figure 6:**
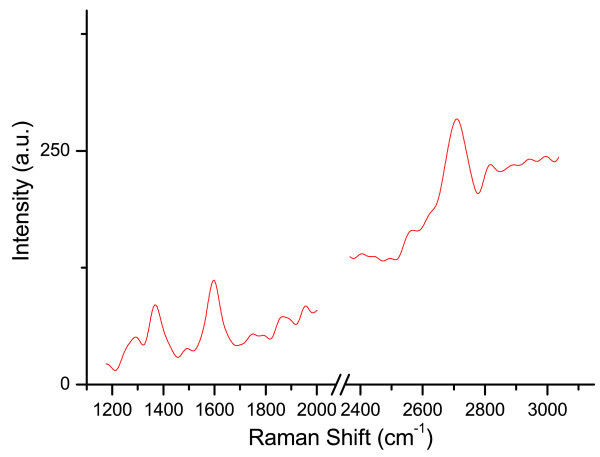
**Raman Spectrum of CVD graphene at low power and long acquisition time.** First Raman spectrum of not transferred CVD graphene grown on Cu, acquired with laser power 0.1 mW for 800 s. The pronounced baseline is typical of graphene Raman spectra acquired on Cu (Miao et al. 2011).

**Figure 7:**
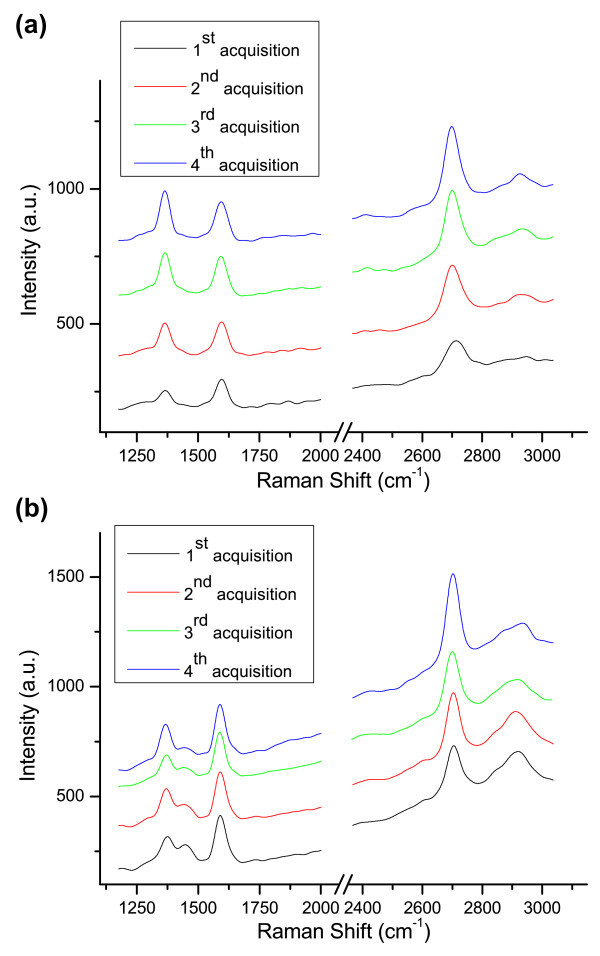
**Raman spectra of CVD graphene at high power and fast acquisition time.** Raman spectra of CVD graphene grown on Cu acquired on the same spot at following time intervals (all the acquisitions have been performed at 1 mW for 80 s): (**a**) graphene onto Cu substrate; (**b**) graphene transferred onto SiO_2_ substrate.

The first spectrum (Figure 6) has been acquired by setting the laser at low power (0.1 mW) and by exposing the sample to laser light for *t* = 800 s. By repeating four times the acquisition in the same spot (of the order of 1 *μ*m^2^) at higher power (1 mW) but reduced exposure time (*t* = 80 s), we obtained the spectra of Figure 7(a). Three prominent peaks are visible: (a) the D peak at ∼ 1340 cm^-1^ associated to a defect induced inter-valley scattering, (b) the G peak at ∼ 1594 cm^-1^ due to in-plane optical vibrations of carbon atoms in the hexagonal crystalline structure and (c) the 2D peak at a Raman shift varying between ∼ 2698 cm^−1^and ∼ 2710 cm^-1^ due to a double-resonant inter-valley scattering involving two in-plane optical phonons (Reina et al. 2009; Tao et al. 2012; Ismach et al. 2010; Su et al. 2011; Malard et al. 2009).

It is possible to notice in particular that while the G peak shape and position are unaffected by laser exposure (only a slight increase in intensity is worthy of note), the D and 2D peaks change significantly their structure.

**Figure 8:**
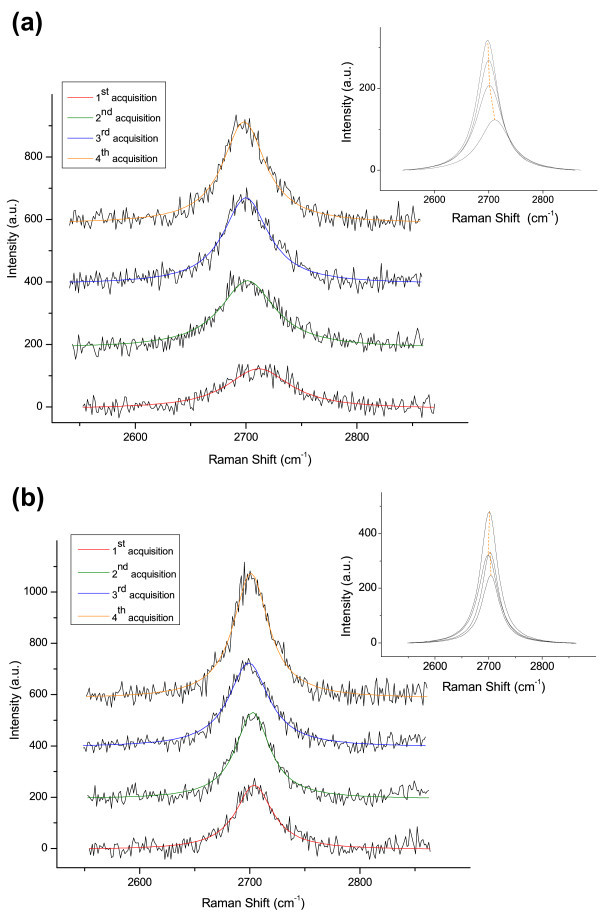
**2D peak evolution with number of Raman acquisitions.** Behaviour of position and shape of 2D peak of Raman spectra acquired in the same spot of the sample: (**a**) graphene onto Cu substrate; (**b**) graphene transferred onto SiO_2_ substrate. (Insets: change in position and intensity of the single Lorentzian curves used to fit the 2D peak of the spectra).

The careful analysis of both the position and the FWHM of the 2D peak for all spectra is reported in Table 1 and Figure 8(a). The results show a significant evolution in the 2D peak shape: this fact may be attributed to some change in the graphene-like structure grown on Cu. The evolution here reported is compatible with a decrease in the number of graphene layers present on the Cu substrate, as suggested by the lowering in the Raman shift position of the peaks centre and by the sharpening of the 2D peaks observed while the exposure to laser light is increased in time. This interpretation is confirmed by the evaluation of the I_G_/I_2D_ ratio of the four spectra: the ratio is decreasing as expected for a decrease in the number of graphene layers (Reina et al. 2009; Ismach et al. 2010; Malard et al. 2009; Ferrari et al. 2006), as shown in Table 1. However, these results do not permit to make a clear quantitative estimation of the number of graphene layers present on the Cu substrate before and after the exposition to the laser light during Raman analysis, because they do not completely agree with the typical values reported in literature for position and FWHM of 2D peak of mono-, bi- and few-layer graphene.

**Table 1:** **2D peak position and sharpness, I**_**G**_**/I**_**2D**_**ratio**

	***Graphene on Cu***			***Graphene on SiO***_***2***_		
Acquisition	Raman Shift	FWHM	I_G_/I_2D_	Raman Shift	FWHM	I_G_/I_2D_
	(cm^-1^)	(cm^-1^)		(cm^-1^)	(cm^-1^)	
1	2711.4	70.0	0.86	2704.0	40.3	1.01
2	2701.3	60.0	0.60	2702.7	37.7	0.79
3	2699.8	49.2	0.56	2699.0	43.8	0.80
4	2698.2	46.6	0.46	2701.1	38.7	0.61

Comparison of the position and FWHM of 2D peaks of subsequent Raman acquisitions as extracted by a single Lorentzian fit and evolution of the I_G_/I_2D_ ratio (spectra from Figure 7, Figure 8). Uncertainties of the order of 0.01 – 0.04% for the 2D peak Raman shifts and of 3 – 6% for 2D peak FHWMs have been estimated. Note: results concerning transferred graphene are affected by a higher uncertainty because of pronounced secondary peaks (possibly due to residual PMMA used for graphene transfer) present in the Raman spectra (see Figure 7(b)), making the fit procedure less accurate.

**Figure 9:**
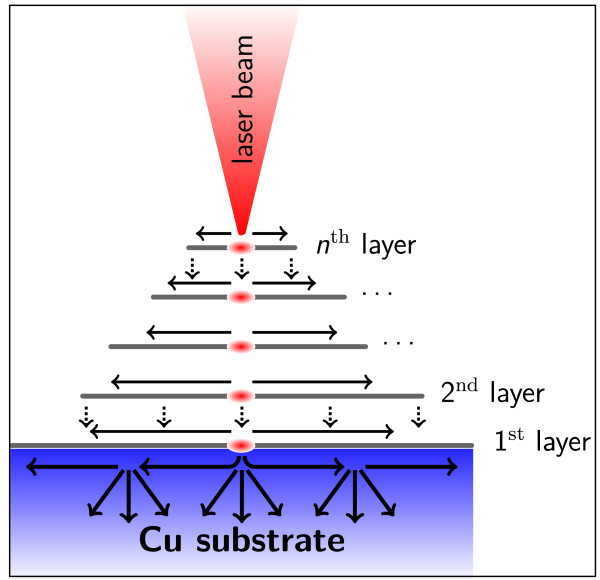
**Phenomenological model for local overheating and etching of multilayer graphene.** Schematic model representing the possible origin of local overheating and etching of outermost graphene layers grown on Cu films. The heat provided by the focused laser beam is dissipated through in-plane (horizontal arrows) and out-of-plane (vertical arrows) channels. Though the in-plane channel is dominant in graphene, the finite size of outer layers makes out-of-plane dissipation significant and reduces the in-plane contribution, thus causing local overheating and subsequent etching of the layers. The innermost layer is instead prevented from etching by the presence of the Cu substrate acting as heat sink.

A possible phenomenological model to explain this effect, as discussed also in (Han et al. 2011), is based on the observation that the laser beam at high power provides an amount of heat sufficient to locally etch away, in presence of oxygen atmosphere, outermost graphene layers grown during CVD process on top of Cu. Although it is known that suspended monolayer graphene shows a room temperature thermal conductivity of up to ∼ 5000 W/(mK) (Balandin et al. 2008), the effect of such a huge value can be significantly reduced in our system because of the decrease in the surface area of graphene flakes occurring while increasing number of layers (said *A*^(*i*)^ the area of the surface covered by the *i*-th graphene flake and *A*^(Cu)^ the area of the underlying Cu grain, *A*^(*n*) ^> *A*^(*n*−1) ^> … > *A*^(2)^ > *A*^(1)^ > *A*^(Cu)^). Moreover, Raman mapping shows different regions with different number of layers on the same sample, corroborating our hypothesis of a terraces-like structure characterizing all the substrate. This fact implies that in-plane heat dissipation through outer graphene layers is largely suppressed by finite-size effects and out-of-plane heat transfer (much lower than the previous one) becomes the dominant heat transfer channel, resulting in a local overheating and subsequent etching of the layers (the model is schematically represented in Figure 9). On the contrary, the innermost graphene layer directly bound to the Cu surface is protected from this effect by the presence of the substrate acting as heat sink. Indeed, it has been reported (Cai et al. 2010) that Cu lowers the thermal conductivity of CVD monolayer graphene grown on it: a significant change in the enhancement of the temperature and in the G peak shifting as a function of the absorbed laser power by using Cu instead of SiO_2_ as a substrate has been reported in this work. As a consequence, graphene-on-Cu and Cu systems have comparable thermal conductivities and the effect of the substrate is not anymore negligible: being Cu a good heat conductor, it provides in turn the dominant contribution to heat dissipation and allows for a more efficient heat exchange through the substrate with respect to the case of transferred graphene onto SiO_2_.

At such photon energies other mechanisms, like molecular desorption of chemical species, cannot be *a priori* excluded, but they can hardly affect the Raman signature and they can be detected through electrical measurements (Sun et al. 2012).

The fact that the G peak position is almost unaffected by the laser irradiation can be explained as a result of two competing effects: the local enhancement of temperature (due to overheating), bringing to a redshift of the G peak (Cai et al. 2010; Calizo et al. 2007) and the decrease in the number of graphene layers (due to etching), resulting instead in a blueshift of the G peak (Wang et al. 2009b).

The unexpected unaltered intensity of the G peak (that should decrease as the number of graphene layers decreases) can be explained in two ways. A first effect, applying to Raman measurements performed both on transferred and not transferred graphene samples, relies on the increase in temperature of graphene layers upon laser irradiation, likely resulting in an effect similar to what observed in experiments concerning the evolution of graphene Raman signature upon controlled annealing at high temperatures (Ni et al. 2008a). As pointed out in this work, the G peak intensity is not changing between few- and monolayer graphene sheets after the annealing process, meaning that in these experimental conditions G peak intensity cannot be regarded as a fingerprint to distinguish number of graphene layers. A second reasoning, applying only on graphene samples over Cu, relies on the roughness of the Cu surface, determining a light trapping effect close to the substrate’s surface that results in an enhanced number of multiple reflections of the laser light between Cu and graphene layers: as a consequence, the number of C atoms detected by the unfocused beam is always comparable to the number of C atoms present in multilayer graphene, although the number of graphene layers is decreasing. As reported in (Ni et al. 2008b) the G band intensity for a number of graphene layers exceeding ∼ 15 is in this case decreasing by increasing number of layers and then constant, as observed in our spectra.

The origin of the prominent D peak (increasing in intensity as the number of acquisitions increases) is not completely clear yet: it can be attributed to the acquisition of the spectra on a point of the sample lying on a grain boundary of the Cu substrate (resulting in a change of the crystallographic orientation of graphene flake through it) or to defects (terrace boundaries) produced in graphene crystal structure by the laser etching.

Raman analysis performed on transferred graphene samples using the same experimental setup (laser at 442 nm, power *P* = 1 mW and exposure time *t* = 80 s) confirms the behaviour observed in the spectra acquired on graphene/Cu substrates. A similar evolution in shape and position of the peaks is obtained for subsequent spectrum acquisitions, as shown in Figure 7(b) and Figure 8(b).

## Conclusions

In summary, a possible way to locally etch graphene layers based on laser heating released during Raman analysis, has been presented. Graphene structure (crystallization degree and number of layers) evolution can be monitored and inferred by looking at the Raman spectra acquired on the sample. The technique is suitable in particular for etching layers of graphene grown by CVD on a metal catalyst. In our case we have reported results obtained with Cu: by lightening the sample with incident laser light at quite high power (≳ 1mW) for short time periods (∼ 80 s) a clear sharpening and lowering of the 2D peak position is observed in Raman spectra, together with a decrease in the I_G_/I_2D_ ratio. These results are compatible with a decrease in the number of graphene layers grown on the metallic substrate. D peak increases in intensity as function of the laser exposure, meaning an increasing of defects in the graphene structure.

We believe that the method can be easily applied to other metallic substrates: since the technique deeply relies on the dispersion of the heat provided by laser irradiation through the substrate, the most important feature required for the substrate is its high thermal conductivity.

Although some catalysts, like Cu, are very promising for CVD synthesis of graphene because of the expected self-limited mono-layered growth of this material on them, a few-layered structure is often found in experiments. Laser etching here reported can therefore provide an *in-situ* technique to get rid of this problem. However, laser can also have an active rôle in inducing unwanted defects, such as vacancies in pristine graphene films: for this reason the proposed method shall be further developed. We finally envisage the application of the photo-etching process here reported to large areas if efficient and uniform illumination conditions, as those used in our RTA system, are employed.

## Abbreviations

CVD: Chemical vapour deposition
SEM: Scanning electron microscopy
STM: Scanning tunneling microscopy
RMS: Root mean square
AFM: Atomic force microscopy
RTA: Rapid thermal annealing
PMMA: poly(methyl-methacrylate)
XRD: X-Ray diffraction.

## References

[CR1_116] Bae S Kim H Lee Y Xu X Park JS Zheng Y Balakrishnan J Lei T Kim HR Song YI Kim YJ Kim KS Özyilmaz B Ahn JH Hong BH Iijima S Roll-to-roll production of 30-inch graphene films for transparent electrodes Nat Nanotechnol 2010 5 574 578 10.1038/nnano.2010.132 20562870

[CR2_116] Balandin AA Ghosh S Bao W Calizo I Teweldebrhan D Miao F Lau CN Superior thermal conductivity of single-layer graphene Nano Lett 2008 8 902 907 10.1021/nl0731872 18284217

[CR3_116] Cai W Moore AL Zhu Y Li X Chen S Shi L Ruoff RS Thermal transport in suspended and supported monolayer graphene grown by chemical vapor deposition Nano Lett 2010 10 1645 1651 10.1021/nl9041966 20405895

[CR4_116] Calizo I Balandin AA Bao W Miao F Lau CN Temperature dependence of the raman spectra of graphene and graphene multilayers Nano Lett 2007 7 2645 2649 10.1021/nl071033g 17718584

[CR5_116] Campos LC Manfrinato VR Sanchez-Yamagishi JD Kong J Jarillo-Herrero P Anisotropic etching and nanoribbon formation in single-layer graphene Nano Lett 2009 9 2600 2604 10.1021/nl900811r 19527022

[CR6_116] Castro Neto AH Guinea F Peres NMR Novoselov KS Geim AK The electronic properties of graphene Rev Mod Phys 2009 81 109 162 10.1103/RevModPhys.81.109

[CR7_116] Coraux J N’Diaye AT Busse C Michely T Structural coherency of graphene on Ir(111) Nano Lett 2008 8 565 570 10.1021/nl0728874 18189442

[CR8_116] Datta SS Strachan DR Khamis SM Charlie Johnson AT Crystallographic etching of few-layer graphene Nano Lett 2008 8 1912 1915 10.1021/nl080583r 18570483

[CR9_116] de Heer WA Berger C Wu X First PN Conrad EH Li X Li T Sprinkle M Hass J Sadowski ML Potemski M Martinez G Epitaxial graphene Solid State Commun 2007 143 92 100 10.1016/j.ssc.2007.04.023

[CR10_116] Di C Wei D Yu G Liu Y Guo Y Zhu D Patterned graphene as source/drain electrodes for bottom-contact organic field-effect transistors Adv Mater 2008 20 3289 3293 10.1002/adma.200800150

[CR11_116] Elias DC Nair RR Mohiuddin TMG Morozov SV Blake P Halsall MP Ferrari AC Boukhvalov DW Katsnelson MI Geim AK Novoselov KS Control of graphene’s properties by reversible hydrogenation: evidence for Graphane Science 2009 323 610 613 10.1126/science.1167130 19179524

[CR12_116] Emtsev KV Bostwick A Horn K Jobst J Kellogg GL Ley L McChesney JL Ohta T Reshanov SA Röhrl J Rotenberg E Schmid AK Waldmann D Weber HB Seyller T Towards wafer-size graphene layers by atmospheric pressure graphitization of silicon carbide Nat Mater 2009 8 203 207 10.1038/nmat2382 19202545

[CR13_116] Ferrari AC Meyer JC Scardaci V Casiraghi C Lazzeri M Mauri F Piscanec S Jiang D Novoselov KS Roth S Geim AK Raman spectrum of graphene and graphene layers Phys Rev Lett 2006 10.1103/PhysRevLett.97.187401 17155573

[CR14_116] Gilje S Han S Wang M Wang KL Kaner RB A chemical route to graphene for device applications Nano Lett 2007 7 3394 3398 10.1021/nl0717715 17944523

[CR15_116] Han GH Chae SJ Kim ES Güneş F Lee IH Lee SW Lee SY Lim SC Jeong HK Jeong MS Lee YH Laser thinning for monolayer graphene formation: heat sink and interference effect. ACS Nano 2011 263 268 10.1021/nn1026438 21174409

[CR16_116] de Heer WA Conrad EH Hass J The growth and morphology of epitaxial multilayer graphene J Phys-Condens Mat 2008 20 323202(1) 323202(27)

[CR17_116] Ismach A Druzgalski C Penwell S Schwartzberg A Zheng M Javey A Bokor J Zhang Y Direct chemical vapor deposition of graphene on dielectric surfaces Nano Lett 2010 10 1542 1548 10.1021/nl9037714 20361753

[CR18_116] Kim H Mattevi C Calvo MR Oberg JO Artiglia L Agnoli S Hirjibehedin CF Chhowalla M Saiz E Activation energy paths for graphene nucleation and growth on Cu ACS Nano 2012 6 3614 3623 10.1021/nn3008965 22443380

[CR19_116] Kim KS Zhao Y Jang H Lee SY Kim JM Kim KS Ahn JH Kim P Choi JY Hong BH Large-scale pattern growth of graphene films for stretchable transparent electrodes Nature 2009 457 706 710 10.1038/nature07719 19145232

[CR20_116] Kondo D Sato S Yagi K Harada N Sato M Nihei M Yokoyama N Low-temperature synthesis of graphene and fabrication of top-gated field effect transistors without using transfer processes Appl Phys Expr 2010 3 025102(1) 025102(3)

[CR21_116] Lee Y Bae S Jang H Jang S Zhu SE Sim SH Song YI Hong BH Ahn JH Wafer-scale synthesis and transfer of graphene films Nano Lett 2010 10 490 493 10.1021/nl903272n 20044841

[CR22_116] Lee CG Park S Ruoff RS Dodabalapur A Integration of reduced graphene oxide into organic field-effect transistors as conducting electrodes and as a metal modification layer Appl Phys Lett 2009 95 023304(1) 023304(3)

[CR23_116] Lee C Wei X Kysar JW Hone J Measurement of the elastic properties and intrinsic strength of monolayer graphene Science 2008 321 385 388 10.1126/science.1157996 18635798

[CR24_116] Li X Cai W An J Kim S Nah J Yang D Piner R Velamakanni A Jung I Tutuc E Banerjee SK Colombo L Ruoff RS Large-area synthesis of high-quality and uniform graphene films on copper foils Science2009a 324 1312 1314 10.1126/science.1171245 19423775

[CR25_116] Li X Cai W Colombo L Ruoff RS Evolution of graphene growth on Ni and Cu by carbon isotope labeling Nano Lett2009b 9 4268 4272 10.1021/nl902515k 19711970

[CR26_116] Liu W Chung CH Miao CQ Wang YJ Li BY Ruan LY Patel K Park YJ Woo J Xie YH Chemical vapor deposition of large area few layer graphene on Si catalyzed with nickel films Thin Solid Films 2010 518 S128 S132 10.1016/j.tsf.2009.10.070

[CR27_116] Liu L Ryu S Tomasik MR Stolyarova E Jung N Hybertsen MS Steigerwald ML Brus LE Flynn GW Graphene oxidation: thickness-dependent etching and strong chemical doping Nano Lett 2008 8 1965 1970 10.1021/nl0808684 18563942

[CR28_116] Malard LM Pimenta MA Dresselhaus G Dresselhaus MS Raman spectroscopy in graphene Phys Rep 2009 473 51 87 10.1016/j.physrep.2009.02.003

[CR29_116] Martoccia D Willmott PR Brugger T Björck M Günther S Schlepütz CM Cervellino A Pauli SA Patterson BD Marchini S Wintterlin J Moritz W Greber T Graphene on Ru(0001): a 25×25 supercell Phys Rev Lett 2008 101 126102(1) 126102(4) 10.1103/PhysRevLett.101.126102 18851393

[CR30_116] Mattevi C Kim H Chhowalla M A review of chemical vapor deposition of graphene on copper J Mater Chem 2011 21 3324 3334 10.1039/c0jm02126a

[CR31_116] Miao C Zheng C Liang O Xie YH Mikhailov S Chemical vapor deposition of graphene Physics and applications of graphene - experiments 2011

[CR32_116] Nandamuri G Roumimov S Solanki R Chemical vapor deposition of graphene films Nanotechnology 2010 21 145604(1) 145604(4) 10.1088/0957-4484/21/14/145604 20215663

[CR33_116] Ni ZH Wang HM Ma Y Kasim J Wu YH Shen ZX Tunable stress and controlled thickness modification in graphene by annealing ACS Nano2008a 2 1033 1039 10.1021/nn800031m 19206501

[CR34_116] Ni ZH Wang YY Yu T Shen ZX Raman spectroscopy and imaging of Graphene Nano Res2008b 1 273 291 10.1007/s12274-008-8036-1

[CR35_116] Novoselov KS Geim AK Morozov SV Jiang D Zhang Y Dubonos SV Grigorieva IV Firsov AA Electric field effect in atomically thin carbon films Science 2004 306 666 669 10.1126/science.1102896 15499015

[CR36_116] Obraztsov AN Obraztsov EA Tyurnina AV Zolotukhin AA Chemical vapor deposition of thin graphite films of nanometer thickness Carbon 2007 45 2017 2021 10.1016/j.carbon.2007.05.028

[CR37_116] Paredes JI Villar-Rodil S Tascón JMD Martínez-Alonso A Graphene oxide dispersions in organic solvents Langmuir 2008 24 10560 10564 10.1021/la801744a 18759411

[CR38_116] Peres NMR The transport properties of graphene: an introduction Rev Mod Phys 2010 82 2673 2700 10.1103/RevModPhys.82.2673

[CR39_116] Peres NMR Guinea F Castro Neto AH Electronic properties of disordered two-dimensional carbon Phys Rev B 2006 73 125411(1) 125411(23)

[CR40_116] Reina A Jia X Ho J Nezich D Son H Bulovic V Dresselhaus MS Kong J Large area, few-layer graphene films on arbitrary substrates by chemical vapor deposition Nano Lett 2009 9 30 35 10.1021/nl801827v 19046078

[CR41_116] Sasaki M Yamada Y Ogiwara Y Yagyu S Yamamoto S Moiré contrast in the local tunneling barrier height images of monolayer graphite on Pt(111) Phys Rev B 2000 61 15653 15656 10.1103/PhysRevB.61.15653

[CR42_116] Schniepp HC Li JL McAllister MJ Sai H Herrera-Alonso M Adamson DH Prud’homme RK Car R Saville DA IAv Aksay Functionalized single graphene sheets derived from splitting graphite oxide J Phys Chem B 2006 110 8535 8539 10.1021/jp060936f 16640401

[CR43_116] Somani PR Somani SP Umeno M Planes nano-graphenes from camphor by CVD Chem Phys Lett 2006 430 56 59 10.1016/j.cplett.2006.06.081

[CR44_116] Sprinkle M Siegel D Hu Y Hicks J Tejeda A Taleb-Ibrahimi A Le Févre P Bertran F Vizzini S Enriquez H Chiang S Soukiassian P Berger C de Heer WA Lanzara A Conrad EH First direct observation of a nearly ideal graphene band structure Phys Rev Lett 2009 103 226803(1) 226803(4) 10.1103/PhysRevLett.103.226803 20366119

[CR45_116] Starr DE Pazhetnov EM Stadnichenko AI Boronin AI Shaikhutdinov SK Carbon films grown on Pt(111) as supports for model gold catalysts Surf Sci 2006 600 2688 2695 10.1016/j.susc.2006.04.035

[CR46_116] Su CY Lu AY Wu CY Li YT Liu KK Zhang W Lin SY Juang ZY Zhong YL Chen FR Li LJ Direct formation of wafer scale graphene thin layers on insulating substrates by chemical vapor deposition Nano Lett 2011 11 3612 3616 10.1021/nl201362n 21834558

[CR47_116] Sun PZ Zhu M Wang KL Zhong ML Wei JQ Wu DH Cheng Y Zhu HW Photoinduced molecular desorption from graphene films Appl Phys Lett 2012 101 053107(1) 053107(4)

[CR48_116] Tao L Lee J Chou H Holt M Ruoff RS Akinwande D Synthesis of high quality monolayer graphene at reduced temperature on hydrogen-enriched evaporated copper (111) films ACS Nano 2012 6 2319 2325 10.1021/nn205068n 22314052

[CR49_116] Varchon F Feng R Hass J Li X Ngoc Nguyen B Naud C Mallet P Veuillen JY Berger C Conrad EH Magaud L Electronic structure of epitaxial graphene layers on SiC: effect of the substrate Phys Rev Lett 2007 99 126805(1) 126805(4) 10.1103/PhysRevLett.99.126805 17930540

[CR50_116] Wang X Li X Zhang L Yoon Y Weber PK Wang H Guo J Dai H N-Doping of graphene through electrothermal reactions with ammonia Science2009a 324 768 771 10.1126/science.1170335 19423822

[CR51_116] Wang H Wang Y Cao X Feng M Lan G Vibrational properties of graphene and graphene layers J Raman Spectrosc2009b 40 1791 1796 10.1002/jrs.2321

[CR52_116] Zhang Y Tan YW Stormer HL Kim P Experimental observation of the quantum hall effect and Berry’s phase in graphene Nature 2005 438 201 204 10.1038/nature04235 16281031

